# Study of Electrochemical Behavior and a Material Removal Mechanism During Electrolytic Plasma Polishing of 316L Stainless Steel

**DOI:** 10.3390/ma18061307

**Published:** 2025-03-16

**Authors:** Gangqiang Ji, Longfei Ma, Sunan Zhang, Juan Zhang, Liyun Wu

**Affiliations:** 1 Engineering Training Center, Taiyuan Institute of Technology, Taiyuan 030008, China; 2 Northern Shanxi Machinery Manufacturing Limited Liability Company, Taiyuan 030009, China

**Keywords:** stainless steel, electrolytic plasma polishing, electrochemical behavior, material removal mechanism

## Abstract

Electrolytic plasma polishing technology is widely used in medical devices, aerospace, nuclear industry, marine engineering, and other equipment manufacturing fields, owing to its advantages of shape adaptability, high efficiency, good precision, environmental protection, and non-contact polishing. However, the lack of in-depth research on the material removal mechanism of the electrolytic plasma polishing process severely restricts the regulation of the process parameters and polishing effect, leading to optimization and improvement by experimental methods. Firstly, the formation mechanism of passivation film was revealed based on an analysis of the surface morphology and chemical composition of stainless steel. Subsequently, the dissolution mechanism of the passivation film was proposed by analyzing the change in the valence state of the main metal elements on the surface. In addition, the surface enclosure leveling mechanism of electrolytic plasma polishing (EPP) for stainless steel was proposed based on a material removal mechanism model combined with experimental test methods. The results show that EPP significantly reduces the surface roughness of stainless steel, with Ra being reduced from 0.445 µm to 0.070 µm. Metal elements on the anode surface undergo electrochemical oxidation reactions with reactive substances generated by the gas layer discharge, resulting in the formation of passivation layers of metal oxides and hydroxides. The passivation layer complexes with solvent molecules in the energetic plasma state of the gas layer with SO_4_^2−^ ions, forming complexes that enter the electrolyte. The dynamic balance between the formation and dissolution of the passivation film is the key to achieving a flat surface. This study provides theoretical guidance and technical support for the EPP of stainless steel.

## 1. Introduction

Electrolytic plasma polishing (EPP) represents a novel non-traditional surface polishing technology that is widely applied in the finishing of stainless steel, cobalt-chromium alloys, titanium alloys, nickel-based alloys, copper alloys, and other metals and alloys [1,2,3,4,5,6]. The most significant advantage of this technology is the use of an environmentally benign inorganic salt solution as the electrolyte, which does not generate toxic byproducts during the polishing process and is highly malleable to the shape of the component. Compared to mechanical polishing, chemical polishing, and electrochemical polishing, it offers several advantages. These include high processing efficiency, superior surface quality after processing, the prevention of macroscopic stress introduction, the absence of microcracks during the polishing process, and a comprehensive range of polishable materials [7,8,9]. It has been demonstrated that electrolytic plasma polishing results in a notable reduction in the surface roughness values of the metal components. This improvement in machining consistency is accompanied by an enhanced gloss and considerable enhancement in corrosion resistance, which can be attributed to the passivation of the surface [10,11].

The material removal mechanism of electrolytic plasma polishing has also been extensively investigated as a theoretical basis for the study of polishing technology [12,13]. The prevailing theoretical framework is based primarily on the gas discharge theory of physical and chemical removal. The theory posits that in the micro convex position of the workpiece surface, the local electric field strength is larger, the spacing between the cathode and the anode is smaller, the electron movement speed is faster, and the electrons enter the anode more rapidly [14]. Consequently, these positions leave more space charge, and the electric field generated by them is more likely to reach the critical value of the aberration caused. The probability of forming a discharging channel in these positions is greater than that in the convex position, resulting in a lower probability of forming a discharging channel in the concave position [15,16]. The formation of discharge channels in recessed positions is less probable than in raised positions. This leads to the removal of more material in the raised positions than in the recessed positions, thereby achieving microscopic leveling of the entire surface. In the case of stainless steel materials, electrolytic plasma polishing can be defined as a dynamic process in which gas discharge and chemical reactions occur simultaneously. This occurs when the discharge removal rate is greater than the reaction generation rate in polishing mode [17]. From a macroscopic perspective, when the workpiece is submerged in an electrolyte at a specific temperature and concentration, the electrolyte immediately transitions to an electrolytic state. Under the influence of ohmic heat, the electrolyte surrounding the workpiece vaporizes rapidly. The electrolyte, a relatively stable water-vapor-based gas layer, separates the workpiece from the electrolyte. The presence of the gas layer increases the circuit resistance and causes a local high voltage to drop. The gas layer is ionized and discharged under the action of high pressure [18]. The ionization of the gas layer, formation of discharge channels, and generation of plasma are the results of a breakdown discharge. These processes lead to a strong and complex plasma physicochemical reaction between the metal surface and gas layer. This reaction simultaneously generates a chemical reaction product on the metal workpiece and removes it via discharge [19].

Additionally, it is postulated that anodic dissolution is predominantly facilitated by electrochemical and chemical reactions. In the case of an anode that is in a state of electroactivity, the material inevitably undergoes an oxidation reaction and generates a passivation film. The formation of this passivation film reduced the dissolution rate of the electrolytic plasma polishing process. This occurs when the rate of formation of the oxide film is comparable to its rate of removal of the oxide film. At this point, the anode enters the polishing mode. During this process, the surface roughness value of the workpiece is reduced. This differs from the electrochemical polishing reaction at low voltages.

The electrolytic plasma polishing process involves several key stages, including the formation of an anode workpiece, the generation of a vapor gas layer, the introduction of an electrolyte, and the establishment of a plasma interface. These stages give rise to a range of complex energy transfers, material diffusion and migration, plasma electrochemical reactions, and other processes. The electrolytic plasma polishing reaction mechanism of stainless steel serves as a foundation for understanding the material removal process. The study of the surface roughness and material removal rate influencing factors is an essential component of this process. In addition, process parameter optimization and the underlying reasons for the alteration of the polished workpiece surface properties are also significant elements. Furthermore, they play a pivotal role in expanding the applications of polished materials and enhancing the design and development of polishing equipment. Currently, research on the reaction of electrolytic plasma polishing interfaces remains relatively limited. This paper presents a study on the electrochemical reaction during the electrolytic plasma polishing of stainless steel, conducted both experimentally and theoretically. The reaction mechanism model has been established, the material removal mechanism of electrolytic plasma polishing has been elucidated from a microscopic point of view, and the microscopic leveling mechanism of electrolytic plasma polishing has been described. These findings provide a foundation for further research on the electrolytic plasma polishing mechanism of stainless steel.

## 2. Materials and Methods

### 2.1. Sample Preparation

**Figure 1:**
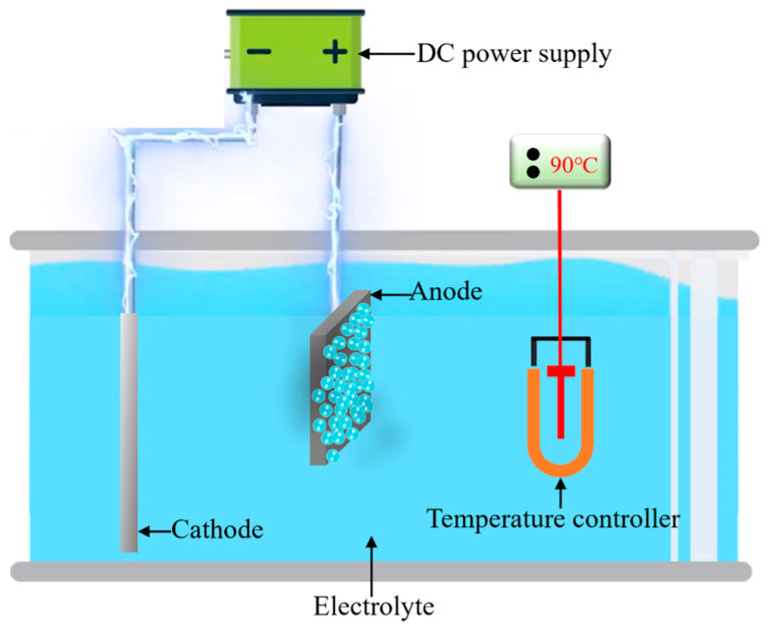
The configuration of electrolytic plasma polishing.

The fundamental configuration of the electrolytic plasma polishing apparatus is shown in Figure 1. It encompasses a power supply system, electrolytic cell, electrode, and electrolyte temperature control system. The material examined in this study was 316L stainless steel (TISCO, Taiyuan, China). The chemical composition of the material is as follows (in wt. %): C (0.03), Cr (18.5), Ni (12.7), Mo (2.10), Mn (2.00), P (0.035), S (0.02), Si (0.80), and the remainder is Fe. The sample was cut into smaller pieces, with dimensions of 20 mm × 15 mm × 3 mm, following the wire-electrode cutting method. The samples were sanded sequentially with 120, 240, and 500 mesh silicon carbide sandpapers (Xili, Wuhan, China) to obtain a consistent untreated surface. Following the pretreatment, the mean arithmetic roughness Ra of the 316L stainless steel surface was found to be approximately 0.44 μm. The electrolyte was a 3% (wt. %) ammonium sulfate (Kermel, Jinan, China) aqueous solution, the polishing voltage was set at 300 V, the electrolyte temperature was set to 90 ± 3 °C, and the processing time was set to 10 min. Once the polishing process was complete, the sample was rinsed multiple times with anhydrous ethanol (Kermel, Jinan, China) and deionized water. This was performed to remove any residual electrolytes that may have remained on the surface.

### 2.2. Characterization and Measurement

The surface profile roughness of the samples was taken as the evaluation index of electrolytic plasma polishing with respect to the product geometry technical specification GB/T 3505-2009 (ISO 4287:1997) and the terms, definitions, and technical specifications of the surface structure. The measurement index was taken as the contour arithmetic mean roughness Ra, root-mean-square roughness Rq, and average maximum height Rz. The measuring equipment used was a high-precision surface roughness measuring instrument (Mahr M400, Gottingen, Germany). It has a resolution of 0.8 nm and a contact measuring force of 0.75 mN. The three-dimensional morphology and average surface roughness Sa of the surface were quantified using an ultra-depth field microscope (Leica DM6M, Wetzlar, Germany). The surface morphology was characterized by atomic force microscopy (AFM, Bruker Dimension Icon, Billerica, MA, USA). The surface elemental distribution was characterized using an energy dispersive spectrometer (Oxford X-MaxN50, Oxford, UK) and a field emission scanning electron microscope (FE-SEM, JSM-7200F, Tokyo, Japan), at an accelerating voltage of 20 kV and a step size of 0.1 μm. The surface chemical characteristics of the samples were characterized by X-ray photoelectron spectroscopy (XPS, Thermo Scientific K-Alpha, Waltham, MA, USA). The analysis was conducted in a vacuum of 8.9 × 10^10^ Pa, with an excitation source of an Al-K x-ray beam (HV = 1486.6 eV), an operating voltage of 12.5 kV, a filament current of 16 mA, and a signal accumulation of 10 cycles. The test was passed with an energy of 50 eV in the full spectrum and 20 eV in the narrow spectrum, with a step size of 0.05 eV and a residence time of 40–50 ms. The chemical states of Fe 2p, Cr 2p, and Ni 2p were referred to the information in the XPS data manual and website. The charge correction was conducted by the C1s = 284.80 eV binding energy standard. Avantage software (V5.52) was employed for the fitting analysis of the obtained data.

## 3. Results and Discussion

### 3.1. Surface Roughness and Morphology

**Figure 2:**
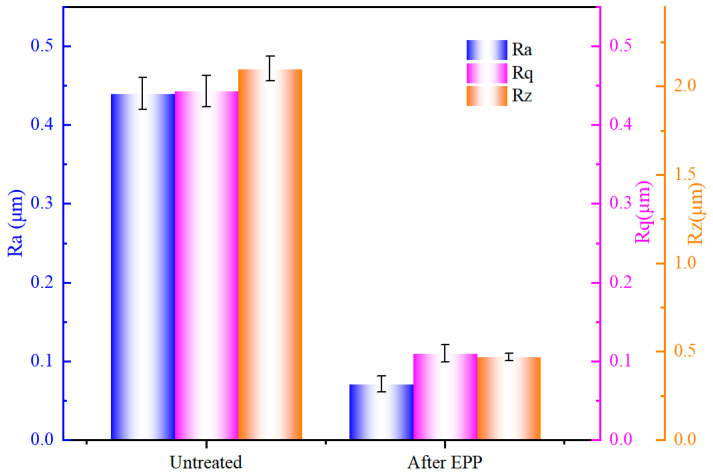
Surface roughness parameters Ra, Rq, and Rz before and after polishing.

Figure 2 illustrates the variation of surface roughness parameters Ra, Rq, and Rz before and after polishing. It can be seen that the surface roughness values decreased significantly after EPP, Ra decreased from the untreated 0.445 µm to 0.070 µm, Rq decreased from 0.445 µm to 0.104 µm, and Rz decreased from 2.147 µm to 0.4711 µm, with a reduction rate of 84.25%, 76.64%, and 78.09%, respectively. The findings indicate that EPP has the potential to significantly diminish the surface roughness of stainless steel materials and enhance surface smoothness and cleanliness.

**Figure 3:**
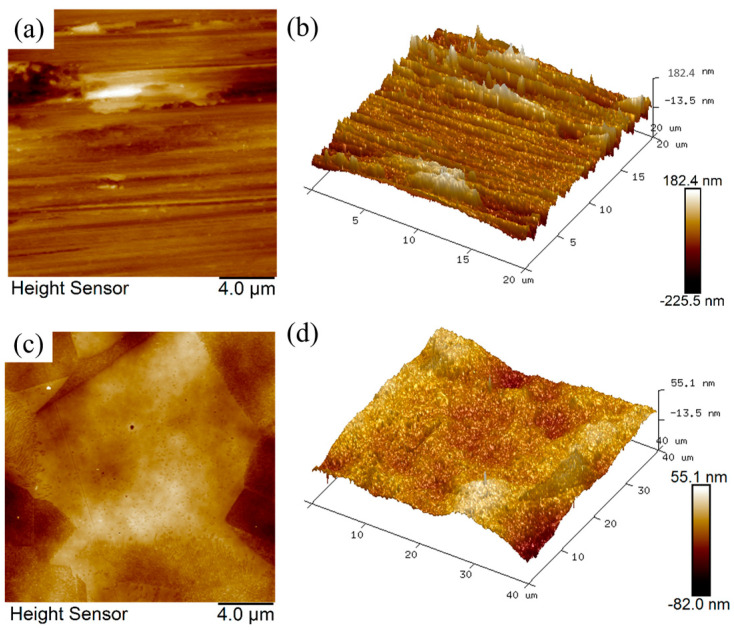
AFM images and 3D topographies of the samples: (**a**,**b**) untreated; (**c**,**d**) after EPP.

The AFM microscopic images and 3D topography of 316L SS samples before (untreated) and after EPP are shown in Figure 3, with scanned areas of 20 × 20 µm and 40 × 40 µm, respectively. The AFM image demonstrates that the unpolished surface is characterized by a rough and uniform texture comprising scratches, bumps, and pits, which are a consequence of the pretreatment process. The polished surface was characterized by a smooth texture and complete removal of traces of scratches, bumps, and pits. In addition, the grain boundary structure is discernible. As illustrated in the 3D topographic map, the elevation difference between the peaks and valleys on the unpolished surface was readily apparent, with discernible scratches. In contrast, the peak and valley heights of the EPP-treated surface exhibited a notable reduction and more uniform distribution. The average surface roughness Sa was reduced from 0.62 µm to 0.084 µm. The findings demonstrate that EPP can effectively eliminate the bumps on the surface of 316L stainless steel, imparting a more uniform distribution of surface peaks and valleys and improving the surface topography.

### 3.2. Elemental Analysis of Material Surface Layers

**Figure 4:**
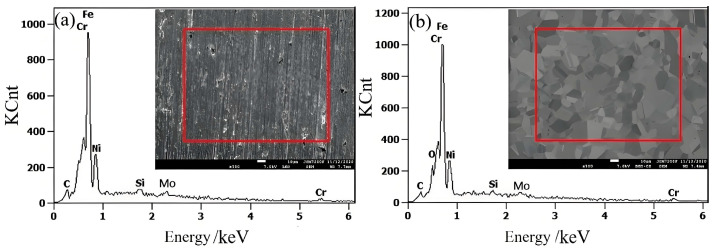
The EDS spectra of stainless steel samples: (**a**) untreated; (**b**) EPP.

The EDS patterns of 316L stainless steel samples before and after electrolytic plasma polishing are shown in Figure 4, and the weight percentage distributions of the major elements are presented in Table 1. The C and Mo content remained essentially unchanged before and after the polishing process. In contrast, Si displayed a notable decline, while Cr exhibited a reduction of approximately 3% after polishing. This may be attributed to the precipitation of elevated Cr concentrations along the grain boundaries, which formed oxides during the EPP procedure. The Ni and Fe elements exhibited an increase in concentration compared to the pre-polishing period. This is attributed to the electrochemical dissolution that occurs during electrolytic plasma polishing, whereby impurity elements are removed from the surface of the 316L stainless steel sample. The reduction in Cr led to a relative increase in the concentrations of Fe and Ni.

**Figure 5:**
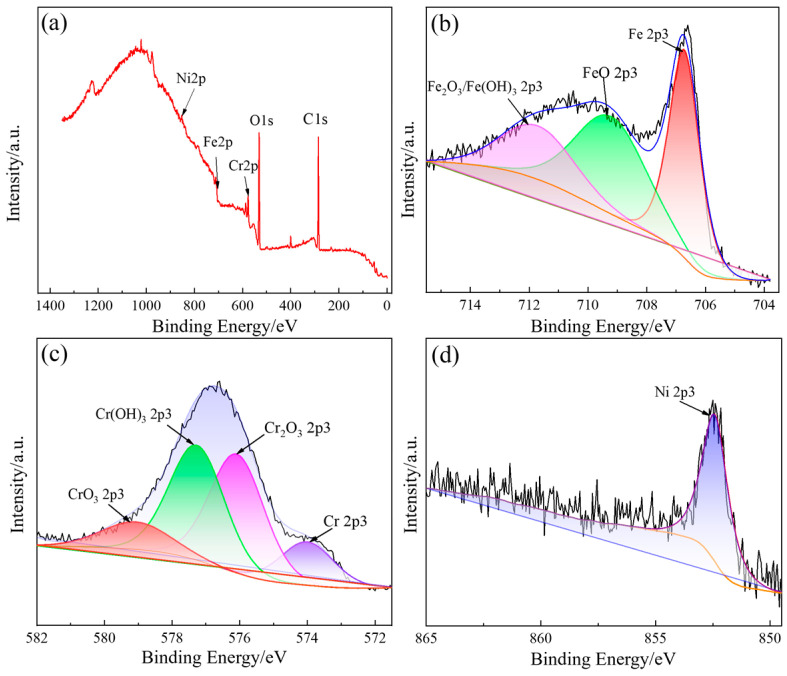
The XPS spectrum of surface elements on 316L stainless steel: (**a**) wide spectrum; (**b**) fine spectrum of Fe2p3; (**c**) fine spectrum of Cr2p3; (**d**) fine spectrum of Ni2p3.

The XPS spectra of the main metal elements on the surface of 316L stainless steel after electrolytic plasma polishing are shown in Figure 5. Figure 5a shows the complete XPS spectrum of 316L stainless steel, wherein the distinctive peaks of Ni2p3, Fe2p3, Cr2p3, O1s, and C1s are distinctly discernible. The Ni2p3 peak is situated at approximately 852.5 eV, the Fe2p3 peak is situated at approximately 707 eV, the Cr2p3 peak is situated at approximately 577 eV, the O1s peak is situated at approximately 532 eV, and the C1s peak is situated at approximately 284.8 eV. To ascertain the precise composition and relative abundance of the Fe, Ni, and Cr elements, the XPS fine spectrum was subjected to peak fitting using Avantage software.

As illustrated in Figure 5b, the XPS fine spectrum of Fe2p3 on the surface of the sample reveals the presence of three peaks, each with a corresponding energy value of 706.7 eV. These peaks can be attributed to the following elements: monolithic iron, FeO, and Fe_2_O_3_/Fe(OH)_3_, respectively. The atomic percent composition of the surface of the polished 316L stainless steel is 37.69%, 39.88%, and 22.43%, respectively. The iron element on the surface of 316L stainless steel after polishing exists in the form of monovalent iron, divalent iron, and trivalent iron. The ratio of divalent iron to trivalent iron is 1.78:1. The formation of iron oxides and hydroxides is primarily the result of electrochemical action during the polishing process.

Figure 5c shows the XPS fine spectrum of Cr2p3 on the sample surface. The Cr2p3 spectrum on the surface of 316L stainless steel after polishing comprises four distinct peaks. These peaks are indicative of the presence of monoprotic chromium at 574 eV, Cr_2_O_3_ at 576.1 eV, Cr(OH)_3_ at 577.3 eV, and CrO_3_ at 579.1 eV; the atomic percent contents of these peaks are 13.54%, 38.11%, 37.25%, and 11.09%, respectively. It is evident that a proportion of the chromium present on the sample surface was engaged in the anodic oxidation reaction during the polishing process, manifesting as monomorphic chromium, trivalent chromium, and hexavalent chromium, with a ratio of the three contents of 1.22:6.8:1. This observation suggests that the primary oxidation products of chromium are Cr_2_O_3_ and Cr(OH)_3_.

Figure 5d depicts the XPS fine spectrum of Ni2p3, which exhibits a single peak at 852.4 eV on the surface of the sample. This value corresponds to metallic nickel, which may be attributed to the fact that the elemental nickel oxidizes under a weakly acidic environment, forming ions that then enter the electrolyte. Therefore, the nickel present on the surface layer of the sample is present as a single substance [20,21].

### 3.3. Electrochemical Behavior Analysis

Electrochemical reactions occur at the anode interface during the EPP of stainless steel, including the precipitation of hydrogen. The generation of water vapor and ionization of oxygen under the influence of the gas layer discharge produces strong oxidizing free radicals. The anode surface of metal elements (Fe, Cr, Ni, etc.) is oxidized by highly oxidizable plasma water molecules, resulting in the formation of metal oxides and hydroxide passivation layers [22,23,24]. The oxidation of the aforementioned elements occurs as follows: Fe is oxidized to FeO, Fe_2_O_3_, and Fe(OH)_3_; Cr is oxidized to Cr_2_O_3_, CrO_3_, and Cr(OH)_3_; and Ni may be oxidized to NiO and Ni(OH)_2_, and the possible reaction processes are as follows [25]:

2H_2_O^*^ → 4H^+^ + O_2_ + 4e^−^ (1)

2H_2_O^*^ → H_2_O_2_ + 2H^+^ + 2e^−^ (2)

Fe + H_2_O^*^ → FeO + 2H^+^ + 2e^−^ (3)

FeO +3H_2_O^*^ → Fe(OH)_3_+ 3H^+^ + 3e^−^ (4)

2Fe + 3H_2_O^*^ → Fe_2_O_3_ + 6H^+^ + 6e^−^ (5)

2Cr + 3H_2_O^*^ → Cr_2_O_3_ + 6H^+^ + 6e^−^ (6)

Cr + 3H_2_O^*^ →Cr(OH)_3_ + 3H^+^ + 3e^−^ (7)

Cr_2_O_3_ + 3H_2_O^*^ → 2CrO_3_ + 6H^+^ + 6e^−^ (8)

Ni + H_2_O^*^ → NiO + 2H^+^ +2e^−^ (9)

Ni + 2H_2_O^*^ → Ni(OH)_2_ + 2H^+^ + 2e^−^ (10)

The passivation layer generated on the surface of the stainless steel prevents further oxidation of the internal metal elements. Owing to the unevenness of the sample surface, the dissolution of the passivation layer was also uneven. In the presence of a high electric field, the passivation layer on the elevated portion of the material surface forms complexes with sulfate and ammonium ions present in the electrolyte. This results in the generation of soluble sulfate and water complexes, which enter the electrolyte as free ions and combine with the hydroxyl radicals in the solution to regenerate the precipitate. Consequently, the plasma-assisted electrochemical dissolution process of metal oxides and hydroxides on the anode surface can be described as follows [26]:

FeO + 2H^+^ + SO_4_^−2^ + H_2_O → FeSO_4_**·**2H_2_O (11)

Fe_2_O_3_ + 6H^+^ + 3SO_4_^−2^ + H_2_O → Fe_2_(SO_4_)_3_**·**4H_2_O (12)

2Fe(OH)_3_ + 6H^+^ + 3SO_4_^−2^ + H_2_O → Fe_2_(SO_4_)_3_**·**4H_2_O (13)

Fe^2+^ + 3H_2_O^*^ → Fe(OH)_3_ + 3H^+^ + e^−^ (14)

Fe^3+^ + 3OH^−^ → Fe(OH)_3_ (15)

Cr_2_O_3_ + 6H^+^ + 3SO_4_^−2^ + H_2_O → Cr_2_(SO_4_)_3_**·**4H_2_O (16)

2Cr(OH)_3_ + 6H^+^ + 3SO_4_^−2^ + H_2_O → Cr_2_(SO_4_)_3_**·**4H_2_O (17)

Cr^3+^ + 3OH^−^ → Cr(OH)_3_ (18)

NiO + 2H^+^ + SO_4_^−2^ + H_2_O → NiSO_4_**·**2H_2_O (19)

Ni(OH)_2_ + 2H^+^ + SO_4_^−2^ → NiSO_4_**·**2H_2_O (20)

Ni^2+^ + 2OH^−^ → Ni(OH)_2_ (21)

The passivation layer, which is generated by the anodic oxidation reaction during the polishing process, has a dense structure that prevents contact between the base metal and electrolyte. Consequently, the reaction occurs between the passivation layer and electrolyte because the presence of the passivation layer does not affect electron transfer. Therefore, the possible dissolution mechanism of the passivation layer can be summarized as follows [27,28]:

(1) The reaction process in the electrolyte involves the formation of a higher-energy complex between the adsorbed SO_4_^2−^ and the surface of the passivation layer around the metal cation. This complex then dissolved in the electrolyte, resulting in a more reactive area. The anodic electric field was rapidly transferred to the other cations on the surface, with a greater number of SO_4_^2−^ ions in contact with the complex. This promotes the dissolution of the complex into the solution and the electrolyte, and then reacts with OH^−^ in the solution, forming hydroxide precipitation.
(2) The adsorption of SO_4_^2−^ results in the formation of cationic vacancies at the interface between the passivation layer and the electrolyte. If the diffusion rate of the vacancies is greater than the rate of cation generation, the cationic vacancies undergo rapid aggregation, leading to the thinning of the passivation layer or even to the direct peeling of the stainless steel surface.
(3) The plasma generated by the gas layer discharge has the effect of destabilizing the passivation layer. The sulfate ions adsorbed in the vicinity of the anode can pass through the passivation layer under the influence of the high electric field strength at the elevated position. This results in the production of strongly conductive ions. The high current density at elevated positions renders the passivation layer cations chemically active. When the electric field at the interface between the passivation layer and the solution attains a critical value, the passivation layer undergoes dissolution.

### 3.4. Material Removal Mechanism

**Figure 6:**
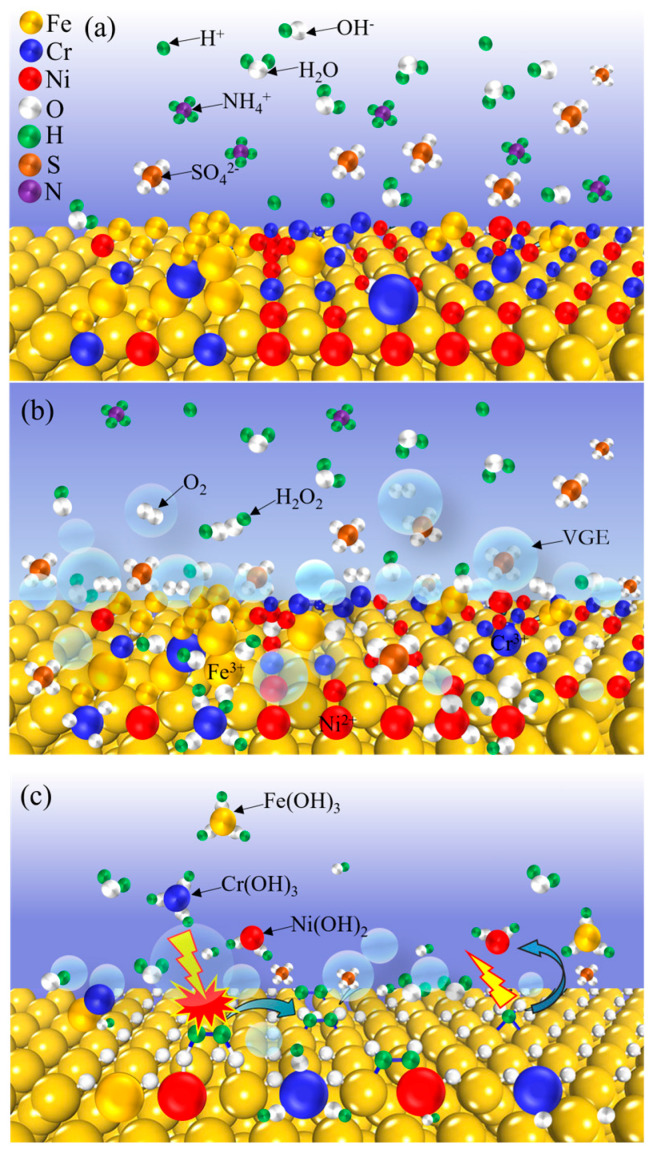
The model of anode interfacial electrochemical reaction mechanism during EPP: (**a**) no voltage applied; (**b**) oxidation reaction; (**c**) dissolution reaction.

The anode interface reactions during electrolytic plasma polishing are coupled with a physical reaction and a multistep plasma electrochemical reaction process. In this study, an anode interface plasma electrochemical reaction material removal mechanism model was established to facilitate a more comprehensive understanding of the reaction process, as illustrated in Figure 6. Figure 6a shows the state of the anode interface when no voltage was applied to the electrode, and the electrolyte was uniformly dispersed with H_2_O, SO_4_^2−^, NH_4_^+^, H^+^ generated by the hydrolysis of NH_4_^+^, and a small amount of OH^−^.

Upon the application of voltage, the anions in the electrolyte are driven towards the anode by an electric field. This process instigates an electrolytic reaction at the anode, leading to the generation of oxygen. Concurrently, the VGE is formed on the surface of the anode through the action of ohmic heat, leading to the generation of free radicals and active substances under the influence of gas layer discharge. This sequence of events is delineated in Figure 6b. At this juncture, the electrolyte and anode surfaces establish contact in the form of an electrolyte bridge, which subsequently separates. When the electrolyte is in contact with the anode, a plasma electrochemical oxidation reaction occurs at the interface of the stainless steel anode. This results in the loss of electrons by the metal atoms on the surface of the stainless steel, which then react with the hydroxyl radicals in the plasma excited state and the oxygen in the free state. The resulting products are metal oxides or hydroxides that are deposited on the surface of the anode to form a passivation layer. The presence of the passivation layer serves to increase the impedance of the anode within the circuit, thereby preventing any further oxidation of the stainless steel anode surface. The continuous generation of bubbles between the electrolyte and anode, in conjunction with the continuous generation and rupture of VGE, gives rise to a significant voltage drop between the workpiece and electrolyte. This phenomenon is accompanied by the breakdown of the gas layer and the occurrence of plasma discharge. At the anode interface, a plasma electrochemical dissolution reaction takes place. Furthermore, the passivation layer on the surface of the stainless steel anode is continuously dissolved and enters the electrolyte as a complex. Moreover, the metal ions present within the passivation layer undergo a combination with hydroxyl radicals or hydroxide ions in the solution, thereby resulting in the generation of hydroxyl radicals or hydroxide ions. As illustrated in Figure 6c, the metal ions present in the passivated layer undergo a chemical reaction with hydroxyl radicals or hydroxide ions present in the solution, resulting in the formation of hydroxide precipitation.

### 3.5. Micro-Leveling Mechanism

**Figure 7:**
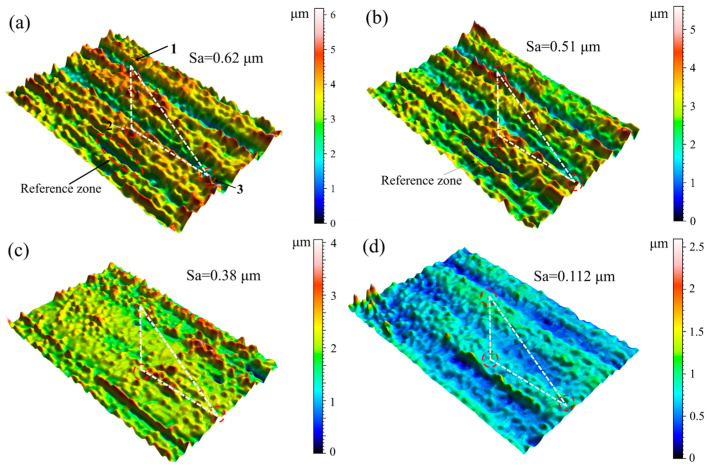
Three-dimensional surface topography of the anode at the same position at different times during EPP: (**a**) 0 s; (**b**) 30 s; (**c**) 120 s; (**d**) 300 s.

The electrolytic plasma polishing process of stainless steel entails the continuous removal of surface material with the influence of an interfacial plasma electrochemical reaction. However, this does not elucidate the macroscopic gradual flattening of the surface. Figure 7 illustrates the evolution of the three-dimensional morphology of the same position of the stainless steel anode at different moments during the electrolytic plasma polishing process.

Figure 7a shows the untreated three-dimensional morphology of the marked position. The left depression was selected as the reference area, and the three highest bumps were selected as the observation positions. The position points were connected to form a localization triangle, thereby enabling the same position at different moments to be determined under the same magnification and the same angle. Figure 7b shows the 3D morphology of the surface at 30 s of polishing. The heights of the three protrusions at the observation positions have decreased by approximately 1–2 μm compared to the untreated position, while the morphology of the depressions remains essentially unchanged. Upon completion of the 120 s polishing period, a notable decrease in the heights of the three bumps at the observation position was observed, accompanied by a progressive flattening of the surface within the observation area. Additionally, the height disparity between the bumps and the depressions diminished, and the number of bumps also declined, as illustrated in Figure 7c. Upon prolonging the polishing process to 300 s, the three marked bumps were largely eliminated, the height disparity between the bumps and depressions in the observation area diminished to approximately 2.5 µm, and the treated region exhibited a uniform and smooth surface, as illustrated in Figure 7d. The surface roughness Sa of the stainless steel samples was reduced from 0.62 µm to 0.112 µm in 300 s. The dissolution rate of the passivation layer on the elevated portion of the material surface is greater than the dissolution rate of the passivation layer on the depressed portion of the surface. As the oxide layer on the elevated portion continues to dissolve, a polishing effect is observed when the dissolution rate exceeds the generation rate of the passivation layer. Consequently, during the electrolytic plasma polishing process of 316L stainless steel, the surface area exhibiting a raised position is consistently and preferentially removed. The continuous reduction in the height differential between the raised and depressed regions leads to a reduction in surface roughness.

## 4. Conclusions

Electrolytic plasma polishing is a complex physicochemical reaction process involving multiphase interfacial reactions. Its interfacial electrochemical reaction mechanism serves as the theoretical basis for the study of the removal of electrolyte plasma-polished materials. In this study, a materials removal mechanism and microscopic leveling mechanism during the electrolytic plasma polishing of stainless steel were investigated from plasma electrochemical oxidation reaction and plasma electrochemical dissolution reaction, respectively, based on an electrochemical reaction. The main conclusions are as follows:

(1) During the electrolytic plasma polishing process of stainless steel, the metal elements on the anode surface and the active substances generated by the gas layer discharge undergo electrochemical oxidation reactions, generating a passivation layer dominated by metal oxides and hydroxides, in which the iron element exists in the form of monomers, FeO, Fe_2_O_3_/Fe(OH)_3_, the chromium element exists in the form of monomers, Cr_2_O_3_, Cr(OH)_3_, and CrO_3_ exist, and the element nickel exists in the form of monomers.
(2) The anode surface raised portions of the untreated gas breakdown discharge phenomenon, whereby the electrolyte and solvent water molecules in the gas layer underwent ionization, evolving into a high-energy plasma state. This state was conducive to the promotion of the metal passivation layer and SO_4_^2−^ ions intermediate reaction, which generated metal salt complexes. The subsequent generation of metal ions from these complexes into the electrolyte resulted in the precipitation of hydroxide. This process was instrumental in achieving the dissolution of the passivation layer.
(3) The principal method of removing stainless steel material is the dynamic cycle of interfacial plasma oxidation and plasma electrochemical dissolution. The uneven dissolution of the passivation layer in raised versus recessed positions is the primary cause of the microscopic leveling of the anodized surface.

## Figures and Tables

**Table 1:** Chemical element composition weight of the samples (wt. %).

Element	C K	Si K	Cr L	Ni L	Fe L	Mo L
Untreated	0.35 ± 0.04	0.86 ± 0.09	19.5 ± 1.05	11.44 ± 0.75	66.17 ± 1.68	2.36 ± 0.36
EPP	0.31 ± 0.05	0.53 ± 0.1	16.6 ± 1.22	12.06 ± 0.57	68.62 ± 0.87	2.26 ± 0.29

## Data Availability

The underlying data of this manuscript are available from the corresponding author upon reasonable request. The data are not publicly available due to privacy restrictions.
